# Macaques in China: Evolutionary dispersion and subsequent development

**DOI:** 10.1002/ajp.23142

**Published:** 2020-05-25

**Authors:** Baoguo Li, Gang He, Songtao Guo, Rong Hou, Kang Huang, Pei Zhang, He Zhang, Ruliang Pan, Colin A. Chapman

**Affiliations:** ^1^ Shaanxi Key Laboratory for Animal Conservation College of Life Sciences, Northwest University Xi'an China; ^2^ International Centre of Biodiversity and Primate Conservation Centre Dali University Dali China; ^3^ Center for Excellence in Animal Evolution and Genetics Chinese Academy of Sciences Kunming China; ^4^ Department of Anthropology McGill University Montreal Quebec Canada; ^5^ School of Human Sciences, The University of Western Australia Perth Western Australia Australia; ^6^ Department of Anthropology Center for the Advanced Study of Human Paleobiology, The George Washington University Washington Washington DC; ^7^ School of Life Sciences, University of KwaZulu‐Natal Scottsville Pietermaritzburg South Africa

**Keywords:** Climate and human‐induced distributional shifts, Macaque radiation, Primate conservation, Primate evolution in China

## Abstract

Depicting a taxonomic group's evolutionary trajectory as a function of changes in the geographical landscape and its historical distribution is critical for constructing informed conservation strategies. Based on fossil sites from the Pliocene to the Holocene, and historical records since 1175 AD, we established macaques’ dispersal pathways into and through China. These routes include internal pathways starting from the southeast corner of the Qinghai‐Tibet Plateau and Mts. Hengduan in western China, and the routes through the estuaries of the three major rivers (Yangtze, Yellow, and Pearl). Our results indicate that macaques used the three rivers and avoided the higher elevation of the plateaus to promote their radiation. They occupied the whole mainland and islands in the Pleistocene and experienced shrunken distribution in the Holocene due to climate changes and human‐induced activities. A prominent China‐wide reduction occurred between 1817 and 1917; and a remarkable retraction from central China happened between 1918 and 2018 following further eco‐social development and human expansion in central China, particularly since the second half of the last century. Starting in 1175 there was a restriction of range to higher altitudes, so that macaques have contracted their range to the west, and the Qinghai‐Tibet Plateau and Mts. Hengduan have become an important sanctuary. We predict that if the current climate and human‐induced changes are not reversed by decisive conservation actions, macaques in east and central China will likely be extinct in the near future.

## Research highlights

- Major waterways used by the primates to disperse and radiate in China
- Primate evolution and dispersion in East Asia from the Pliocene to the Early Holocene
- Historically shrunk macaque distribution in China

## Graphical abstract

Dispersal and radiation pathways undergone by the macaques (Macaca) during the processes of colonising East Asia in the Pliocene, Pleistocene and Early Holocene.

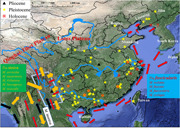

## 1 INTRODUCTION

Broad literature reviews of fossil and extant taxa distributions allow us to have a retrospective reconstruction of animal evolutionary development and historical change. Such efforts allow predictions to be made about species survival prospects and thus are important in reconstructing informed conservation and management strategies (Li, Pan, & Oxnard, 2002; Lotze & Worm, 2009; Turvey, Crees, & Di Fonzo, 2015; Zhao et al., 2018). Historical records of the distribution of sufficient duration are only available for a few civilizations. One of these is that of China with its written history dating back to 1,250 years (Li & Hong, 2007).

**Figure 1:**
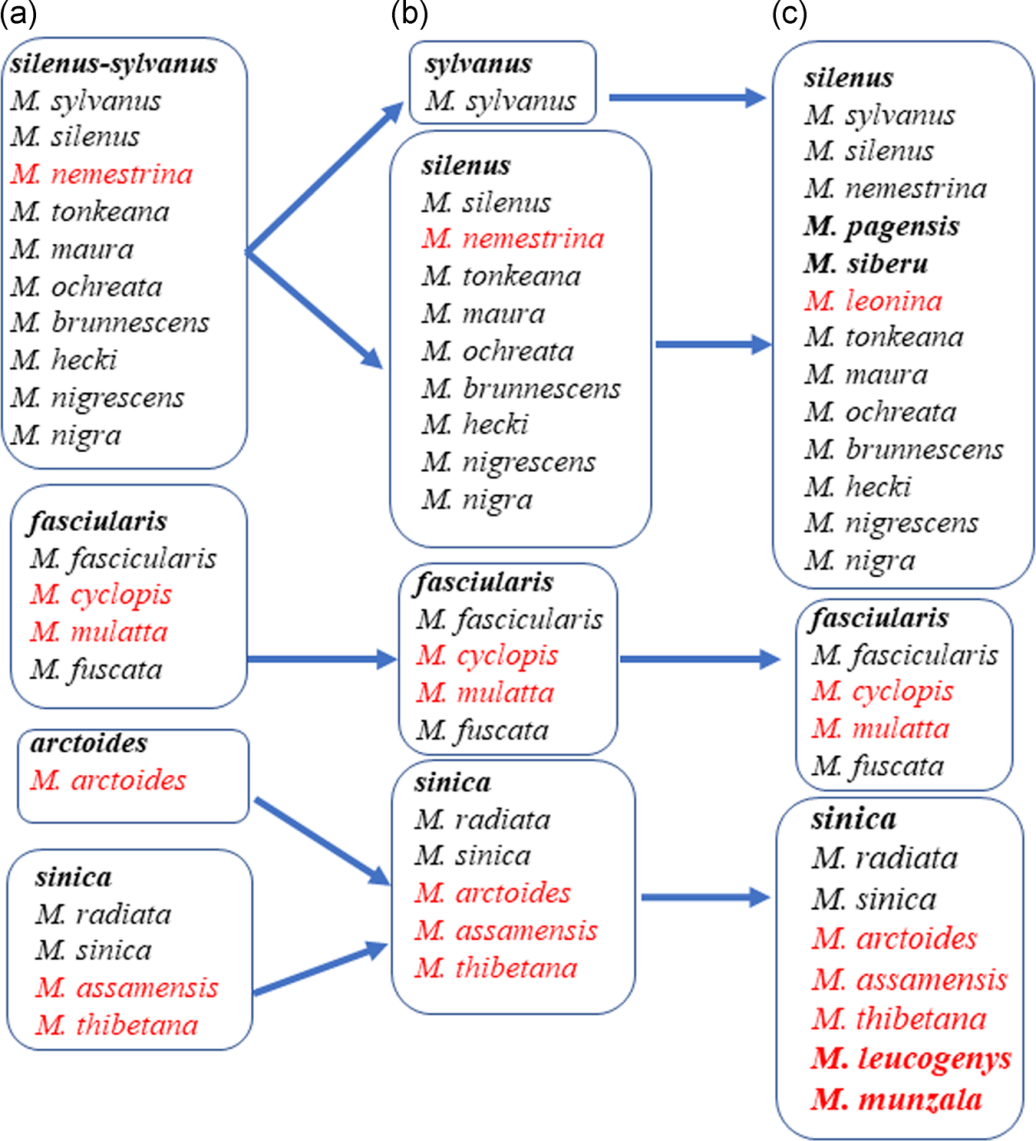
The development of recent classification and phylogeny of the macaques. (a) Fooden (1976) based on the morphology of male reproductive organ; (b) Delson (1980) referring to morphology and geographic distribution: *Macaca sylvanus* found only in North Africa was separated from the *sylvanus‐silenus* group, and *M. arctoides* was combined with the *sinica* group, which is supported by the evidence from Y Chromosome loci (Tosi et al., 2000), Alu elements for genetic markers (Li et al., 2009), and homologous regions of DNA (Deinard & Smith, 2001); and (c) Groves (2000) separated *M. leonina* from *M. nemestrina* based on morphology, which is supported by molecular study (Roos, Thanh, Walter, & Nadler, 2007). *Macaca pagensis* also was separated from *M. nemestrina* (Brandon‐Jones et al., 2004; Tosi et al., 2000). *Macaca siberu*, exclusively on the island of Siberut, the west coast of Sumatra, was separated from *M. pagensis* by Kitchener and Groves (2002) referring to its isolated distribution and morphology. *M. leucogenys* was reported in southeastern Tibet (Li, Zhao, & Fan, 2015), and *M. munzala* was recorded in North Eastern India and Tibet (Sinha et al., 2005). The combination of *M. leucogenys*, and *M. munzala* with the *sinca* group is based on morphology (Li et al., 2015; Sinha et al., 2005), and genetic and molecular evidence (Chakraborty et al., 2007; Fan et al., 2017). Red‐colored species are the taxa found in China, and bolded species are the taxa newly categorized

There are currently 28 species of nonhuman primates in China. Three of them, douc langur (*Pygathrix nemaeus*), and gibbons (*Nomascus leucogenys* and *Hylobates lar*), were recently extirpated, and approximately 80% of these species are endangered (Li et al., 2018; Pan et al., 2016; Zhao et al., 2018). Among them, eight macaque species (*Macaca*) are the most widely distributed. Evolutionarily and phylogenetically, Asian macaques are closely related to the Barbary macaques *(M. sylvanus*) of northern Africa (Delson, 1975; Stewart & Disotell, 1998). They initiated their journey to Asia in the Late Miocene and reached East Asia about 3 Ma ago (Stewart & Disotell, 1998). Varying dispersal and radiation scenarios have been proposed for macaques by different scholars. According to some (e.g., Abegg & Thierry, 2002; Delson, 1980; Fa, 1989), the *silenus*‐*sylvanus* group moved from Africa along the coast to India, Burma, and Malaya, then entered Sundaland, including Borneo and Sumatra. Associated with this movement and subsequent isolation species differentiated into the four species groups listed in Figure 1. Those giving rise to the taxa in China include the *fascicularis* group resulting in the speciation in mainland China, Hainan Island, Taiwan, South Korea, and Japan; the *sinica* group that was thought to have arisen in west Burma from fossil species of *M. anderssoni*. Some taxa in this group are thought to have entered China in the Middle Pleistocene and radiated into what is today *M. thibetana*, *M. arctoides*, *M. assamensis*, *M. leucogenys*, and *M. munzala* (Fan et al., 2017; Mishra & Sinha, 2008; Sinha, Datta, Madhusudan, & Mishra, 2005); and *M. leonina* coming from the *silenus* group originated in the Southeast Asia (Tosi, Morales, & Melnick, 2000).

Such hypothesized pathways are, unfortunately, not schematically backgrounded with geographic landscapes, such as plateaus, and major waterways in China, so that they cannot tell us specifically how a group selected dispersal ways according to the structure of the landscapes.

Climate changes after the Pleistocene and extensive human activities in the Holocene significantly impacted macaque distributions in China (Li et al., 2018; Pan et al., 2016). Currently, *M. mulatta* and *M. cyclopis* are categorized as less concerned (LC), while *M. leonina*, *M. munzala*, *M. arctoides*, *M. thibetana*, and *M. assamensis* are Near Threatened (NT); the status of *M. leucogenys* is undetermined, but it is threatened by the action of local communities (Fan et al., 2017; Zhao et al,  2015). Macaque populations in China are generally more negatively impacted than those outsides because of China's remarkable development that has been dependent principally on the extraction of the natural resources, including extensive deforestation, particularly since the 1950s (Li et al., 2018; Pan et al., 2016).

Here we examine fossil and extant macaque taxa distribution in China to explore the dispersal patterns of *Macaca* during the Pliocene to Holocene, and to predict future distributions following their current changing trajectories. We discuss possible external dispersal routes used by the *sinica* and *fascicularis* groups, and *M. leonina* from the *silenus* groups, into China; propose their within China dispersal routes relying on mainland fossil locations; and display the changes of spatiotemporal distribution trajectories of the macaques in recent history.

## 2 MATERIALS AND METHODS

Our project is based on fossil records and a broad literature review from academic journals, governmental annals, magazines, and books, many written in Chinese and not easily accessible to the international community. We particularly relied on the two by books written in Chinese by Rongsheng Wen (Wen, 2009, 2013; Wen, 2018) that review archives and annals at the county level since 1175 AD, and indicate where large mammals occur. Although different names were given to the same animal groups at different places and historical periods, in most cases this does not influence primate identification (Li et al., 2002; Wen, 2009, 2013). There were four primate types in China: (a) Gibbons called “Yuan” in Chinese, often described by their long upper limbs, slim body structure, and unique calls (Zhang, 2015). Their images have been commonly used in paintings since the Zhou Dynasty (1027–221 BC) (Geissmann, 2008); (b) Snub‐nosed monkeys characterized with up‐turned nostril structure; (c) Leaf‐eating monkeys depending on the forests, and characterized by eating leaves; and (d) Macaques that are primarily terrestrial and morphologically quite different from the other taxa. The unique contribution of macaques to Chinese culture and broad public recognition is well demonstrated by a mythical novel called “*The Journey to the West*” written in Ming Dynasty (1368–1644) (Liu & Li, 2013), which was translated into English in 1943 (Arthur, 1943). In this novel "Sun Wukong," the Monkey King, is an incarnation of the macaques and is also mentioned in Confucianism, Buddhism, and Taoism—"Three‐Religion‐in‐One Ideology" (Liu & Li, 2013; Lou, 2016). We (Li and Pan) validated historical name variations with the methods used in Li et al. (2002).

Although government archives and annals started in China more than 2,000 years ago in the Qin Dynasty (Dai, Liu & Xiao, 2007), the detailed documentation of animal distribution started about 400 years ago (Li et al., 2002). Thus, we used the fossil records, and historical records since 1716 to generate distribution profiles for each century (see Supporting Information Database). Evaluation of the fossil records of Chinese macaques was based mainly on book chapters (Jablonski, 2008), book publications (Lucas, 2001; Wu & Poirier, 1995), and journal papers (e.g., Wu, 2004). Longitudes, latitudes, and altitudes of the locations were determined from Google Earth. Commonly, the same records for a given location in each century were documented in different years and by different publications or archives, only one record was used for mapping. Spatiotemporal distribution maps are generated with ESRI ArcGIS Desktop 10.6, and SPSS Version 20 was used to illustrate altitudinal changes from 1175 AD to the present.

## 3 RESULTS

**Figure 2:**
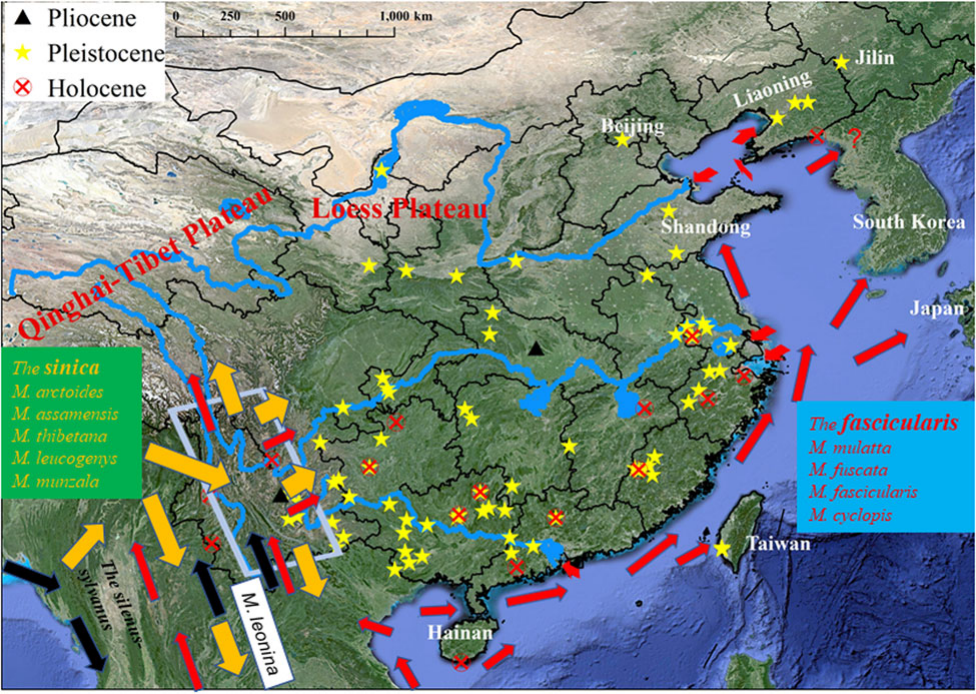
Fossil‐bearing locations of the macaques in China discovered in the Pliocene, Pleistocene, and Holocene (see Supporting Information Database for more details). The dispersal patterns of the *sinica* and *fascicularis* groups, and *M. leonina* are illustrated referring to the hypotheses by Delson (1980), Fa (1989), and Tosi et al. (2003), except for entrances of the three major rivers. The squared area is the southeast corner of the Qinghai‐Tibet Plateau and Mts. Hengduan in Yunnan (SQPMH)

Fossil‐bearing locations of the macaques in China from the Pliocene to Holocene are presented in Figure 2. Among them, the two locations in the Pliocene (the oldest macaque fossils) are in Lufeng, within the Southeast corner of the Qinghai‐Tibet Plateau and Mts. Hengduan (known as the SQPMH), and Zhongxiang in Hubei. The former is older (Upper Pliocene) than the latter (Lower Pliocene). Regarding other fossils, those in the Pleistocene are widely distributed, and except for one at Donggang in Liaoning, all other Holocene fossils are located in the south of the Yangtze River. Among the three major migration waves of macaques in Asia (Abegg & Thierry, 2002; Delson, 1980; Fa, 1989), all of them were directly related to the dispersion and radiation of the macaques in China: the *fascicularis*, the *sinica*, and the *silenus* groups (Figure 2). The *fascicularis* group resulted in the speciation of *M. mulatta* in the Hainan Island and mainland, *M. cyclopis* in Taiwan, and *M. fuscata* in Japan. Some populations of this group, however, moved northward leading to the distribution of *M. mulatta* in Indochina, and west China through the SQPMH; the *sinica* group including five species in China initiated its dispersion from the SQPMH; and *M. leonina* from the *silenus* group moved northward to enter the SQPMH—different from the other macaque species in China, it did not move eastward.

**Figure 3:**
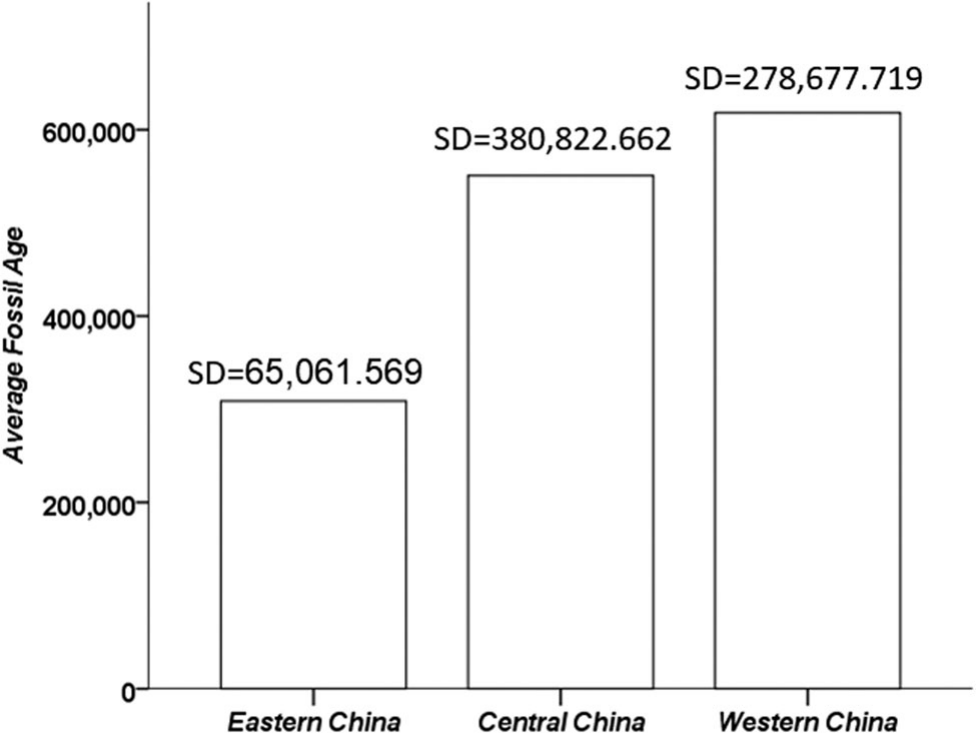
Age differentiation of the fossils among three major Chinese geographic regions. Western China (Chongqing, Gansu, Guizhou, Ningxia, Qinghai, Shaanxi, Sichuan, Xinjiang, Yunnan, and Xizang), Central China (Anhui, Heilongjiang, Henan, Hubei, Hunan, Inner Mongolia, Jiangxi, Jilin, and Shanxi), and Eastern China (Beijing, Fujian, Guangdong, Guangxi, Hainan, Hebei, Jiangsu, Liaoning, Shandong, Shanghai, Tianjin and Zhejiang (Dong & Liang, 2014; Huang, Ma, & Huang, 2017; Wei, 1998). *SD*: standard deviation

The fossil sites show a substantial age differentiation among east, central, and western China (Figure 3). Fossils are progressively younger as one moves eastward in China.

**Figure 4:**
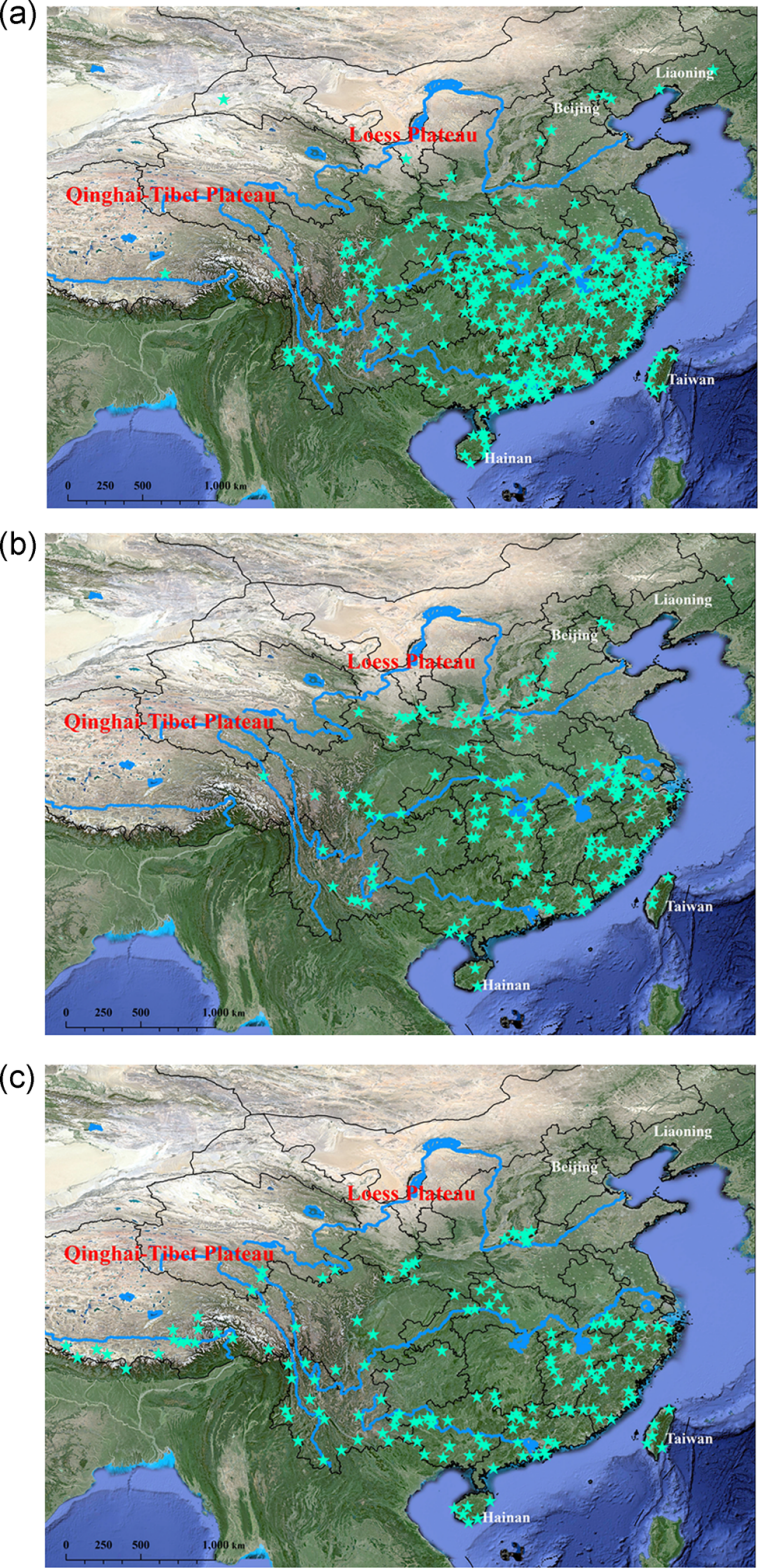
Centurial comparison of chronological distribution profiles of the macaques in China since 1716 (see Supporting Information Database for more details)

**Figure 5:**
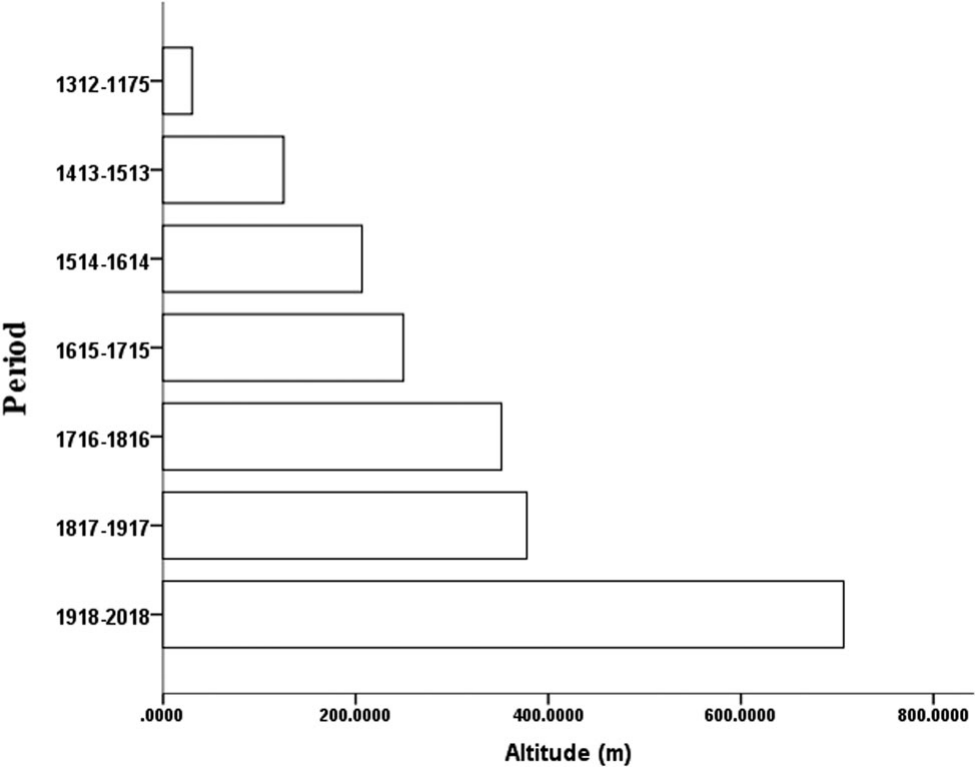
Altitudinal distribution increase of the macaques in China since 1175. The interval between the columns is 100 years, accounting from 2018, so that the last column covers 137 years (1312–1175)

Between 1716 and 1816 historical records suggest that macaques were widely distributed in China, as they were found in the Hainan and Taiwan islands, the northeast corner of the country near Beijing and Liaoning, and in the Qinghai‐Tibet and Loess Plateaus (Figure 4a). Between 1817 and 1917 macaque distribution was generally similar to the previous century, except they were less condensed, particularly in western parts (Figure 4b). Remarkable distribution changes occurred between 1918 and 2018, and many taxa are no longer mentioned in central China. In contrast, several taxa appear for the first time in the Qinghai‐Tibet Plateau (Figure 4c). An altitudinal increase of macaque distribution since 1175 AD is presented in (Figure 5).

## 4 DISCUSSION

Macaques into China in the Pliocene were widely distributed by the Holocene through external and internal routes illustrated in (Figure 2). The fact that all of the Holocene fossils, except one in the northeastern corner (Liaoning), are in south China implies that their distribution prominently changed during the Holocene, particularly moving southward.

### 4.1 External dispersion

The ancestor of Asian macaques separated from their African counterparts in the Miocene between 5.3 and 5.9 Ma (Alba et al., 2014; Delson, 1975; Fa, 1989; Tosi, Morales, & Melnick, 2003). This has been validated based on the evidence from chromosomes and molecular markers (Tosi et al., 2003), gross morphology (Fooden, 1976), fossils (Delson, 1980; Takai et al.,  2014), geographic distribution pattern (Abegg & Thierry, 2002), and genetics (Li & Zhang, 2005; Li et al., 2009; Melnick & Kidd, 1985; Morales & Melnick, 1998). Macaques entered China using two routes; one for the *sinica* group, and *M. leonina* from the *silenus* group through the SQPMH, and the other for the *fascicularis* group along the coastlines of South and East China Seas (Figure 2).

After having reached south India along the coast and spread to Southeast Asia, where the *silenus* group was originated. The *sinica* group, however, dispersed to northern India and approached the Himalayas and Qinghai‐Tibet Plateau (Sakai et al., 2006), which formed a barrier to its further northern dispersion, so that this ancestral group moved eastward into the SQPMH, and subsequently dispersed eastward China (orange arrows in Figure 2), while some moved southward diverging into *M. arctoides* in Cambodia; Laos, Malaysia, Myanmar, Thailand, and Viet Nam (Delson, 1980; Fa, 1989; Fooden, 1982). *M. leonina* from the *silenus* group in the Southeast Asia moved northward (black arrows in Figure 2), and entered into the SQPMH. Interestingly, different from other taxa, this species still remains there, mostly in Western Yunnan Province (Li et al., 2018).

The dispersal procedure of the *fascicularis* group was closely related to climate change; the formation of the glaciation led to the lower sea levels during the Pliocene and Pleistocene, and land bridges formed in East Asia at different times (Voris, 2000). At that time Hainan, Taiwan, and Japan were frequently linked to the mainland until approximately 18,000 years ago (Adams & Faure, 1997). This allowed the diversification and led to *M. mulatta* in Hainan, *M. cyclopis* in Taiwan, and *M. fuscata* in Japan (Fa, 1989). A puzzling question is how *M. mulatta* got into the mainland. That more fossil localities are near the estuaries of the Yangtze, Yellow, and Pearl Rivers in Figure 2 suggests they used the lower parts of the rivers for mainland radiation. The dispersal procedure for the *fascicularis* group is thought to have occurred between 2 and 0.7 Ma (Abegg & Thierry, 2002; Delson, 1980) from Sundaland. On the other hand, the fact that *M. mulatta* is widely distributed in East and South Asia suggests that their ancestors also dispersed northward from the Sundaland (red arrows in Figure 2).

That fossils to the east of China are younger than those in the west suggests that the external dispersal for the *sinica* group may start earlier than the *fascicularis* group (Figure 3). This suggestion is supported by nucleotide research indicating that Taiwan and Japan were colonized around only 0.38–0.44 Ma ago (Chu, Lin, & Wu, 1999).

### 4.2 Internal dispersion

The fact of the oldest fossil‐bearing sites in the Pliocene is in the SQPMH (Figure 2) and western China (Figure 3) implies that macaques entered China through the SQPMH and subsequently dispersed. There appear to be three routes for the procedures: (a) between the Yangtze and Yellow Rivers avoiding the high elevation of the Loess and Qinghai‐Tibet Plateaus The Yellow River formed in the Eocene and originated from the Qinghai‐Tibet Plateau. However, it was substantially modified in the Late Miocene–Early Pliocene due to the orogenic events (Ding & Liu, 1989; Lin et al., 2001). Macaques likely used what is now the Yellow River drainage to cross the river and reached the most northeastern corner of China (Liaoning and Jilin). (b) They also likely used the area between the Yangtze and Pearl Rivers that originate in the SOPMH. During the glaciation in the Late Pleistocene, the water level of the Yangtze River dropped by more than 20 m in some sections (Yang, 1986), thus macaques were likely able to cross the Yangtze. (c) South of the Pearl River contains many macaque fossils suggesting this was also another dispersal route. After having gotten into the mainland through estuaries of the three rivers, populations of the *M. mulatta* started their radiation in China.

### 4.3 Distribution alteration in recent history

Historical records of the last 300 years reveal many changes in macaque distribution in China since 1716 (Figure 4). This was mainly caused by human‐induced activities (Li et al., 2018; Pan et al., 2016). There have been three prominent distribution changes of the macaques over the last three centuries (Figure 4). Compared to the period of 1716–1816, many macaque‐bearing sites were not mentioned between 1817 and 1917 (Figure 4a,b); there were remarkable westward, southward, and eastward shifts between 1918 and 2018. This resulted in a marked disappearance in central China, and the appearance of new taxa appear in the Qinghai‐Tibet Plateau (Figure 4b,c). In addition to climate change (Jablonski & Whitfort, 1999; Zhang et al., 2007), distribution shifts were also associated with economic and social development, particularly its central China (Zhu et al., 2018), such as the cultivated land area increased most quickly in the early Qing Dynasty (Ge et al., 2004). This resulted from technology development and population growth (Lu, 2015), from 388,150,057 in 1799 (Wang, 1997) to about 600 million in 1950 (http://www.china‐profile.com/data/fig_WPP2008_TFR_1.htm). The most remarkable change as shown in Figure 5 was, however, the increased altitudinal scale, resulting in the SQPMH becoming an important sanctuary for them.

China experienced dramatic social and environmental change that is well documented starting from the first dynasty (Shang) more than 3,000 years ago (Wakeman, 1977). Since 1780, China suffered 25 major domestic and international conflicts (Elleman, 2001), including the two Opium Wars in 1834–1842 and 1856–1860 (Feige & Miron, 2008; Hanes & Sanello, 2002). Archives and historical records were likely destroyed or lost during the wars, which may explain some locations not being represented in 1817–1917, but in the previous century (1716–1816). West China, particularly SQPMH, was a sanctuary for plants and animals since the Pleistocene (Jablonski, 1993) and is now a biodiversity spot (Tang et al.,  2006), and contrast east and central China, it is a relatively lower level of human occupancy, so that some macaque taxa moved back to the Qinghai‐Tibet Plateaus and the SOPMH.

## 5 CONCLUSION

Based on the records of fossil‐bearing sites from the Pliocene to Holocene, and historical distribution records since 1175 AD, we evaluated macaque dispersal pathways into and throughout China. The three major waterways in mainland—Yangtze, Yellow, and Pearl Rivers, which originate in the Qinghai‐Tibet Plateau and Mts. Hengduan, have played a key role for monkey dispersion and radiation in China. A significant macaque distribution started to shrink during 1817–1917 period, and there were notable eastward, westward, and southward retractions in the 1918–2018 period, when China experienced rapid economic and social development. Macaques have progressively moved to higher elevation since, and this movement was most rapid in the last century. As a result, west China, particularly the SQPMH, is now for the groups' survival in China, as many populations would be wiped out shortly if current trajectories of development are not curtailed.

## Supporting information

Supporting information

## Data Availability

International Centre of Biodiversity and Primate Conservation, Dali University, and Northwest University, China, http://www.icbpc.org/a/Academic_communication/.
